# TRPV1 Channel Activated by the PGE2/EP4 Pathway Mediates Spinal Hypersensitivity in a Mouse Model of Vertebral Endplate Degeneration

**DOI:** 10.1155/2021/9965737

**Published:** 2021-08-21

**Authors:** Sijing Liu, Qiong Wang, Ziyi Li, Lei Ma, Ting Li, Yukun Li, Na Wang, Chang Liu, Peng Xue, Chuan Wang

**Affiliations:** ^1^Editorial Department of Hebei Medical University, Hebei Medical University, Shijiazhuang, Hebei 050017, China; ^2^Department of Pharmacology, Hebei Medical University, Shijiazhuang, Hebei 050017, China; ^3^Department of Endocrinology, The Third Hospital of Hebei Medical University, Shijiazhuang, Hebei 050051, China; ^4^Key Orthopaedic Biomechanics Laboratory of Hebei Province, Shijiazhuang, Hebei 050051, China; ^5^Department of Spine Surgery, The Third Hospital of Hebei Medical University, Shijiazhuang, Hebei 050051, China; ^6^Institute of Biomedical Engineering, Chinese Academy of Medical Sciences and Peking Union Medical College, Tianjin 300020, China

## Abstract

Low back pain (LBP) is the primary cause of disability globally. There is a close relationship between Modic changes or endplate defects and LBP. Endplates undergo ossification and become highly porous during intervertebral disc (IVD) degeneration. In our study, we used a mouse model of vertebral endplate degeneration by lumbar spine instability (LSI) surgery. Safranin O and fast green staining and *μ*CT scan showed that LSI surgery led to endplate ossification and porosity, but the endplates in the sham group were cartilaginous and homogenous. Immunofluorescent staining demonstrated the innervation of calcitonin gene-related peptide- (CGRP-) positive nerve fibers in the porous endplate of LSI mice. Behavior test experiments showed an increased spinal hypersensitivity in LSI mice. Moreover, we found an increased cyclooxygenase 2 (COX2) expression and an elevated prostaglandin E2 (PGE2) concentration in the porous endplate of LSI mice. Immunofluorescent staining showed the colocalization of E-prostanoid 4 (EP4)/transient receptor potential vanilloid 1 (TRPV1) and CGRP in the nerve endings in the endplate and in the dorsal root ganglion (DRG) neurons, and western blotting analysis demonstrated that EP4 and TRPV1 expression significantly increased in the LSI group. Our patch clamp study further showed that LSI surgery significantly enhanced the current density of the TRPV1 channel in small-size DRG neurons. A selective EP4 receptor antagonist, L161982, reduced the spinal hypersensitivity of LSI mice by blocking the PGE2/EP4 pathway. In addition, TRPV1 current and neuronal excitability in DRG neurons were also significantly decreased by L161982 treatment. In summary, the PGE2/EP4 pathway in the porous endplate could activate the TRPV1 channel in DRG neurons to cause spinal hypersensitivity in LSI mice. L161982, a selective EP4 receptor antagonist, could turn down the TRPV1 current and decrease the neuronal excitability of DRG neurons to reduce spinal pain.

## 1. Introduction

Low back pain (LBP) is the primary cause for disability globally [1], with a 1-month prevalence of 23.2% [2]. Since LBP is generally a persistent symptom, about 2/3 of the patients with LBP complained about their pain-related symptoms even after 12 months [3]. This persistent painful condition is associated with the development of multiple physical and psychosocial disabilities [4]. In 2017, a total of 577 million people experienced LBP, and more than 60 million healthy life years were lost worldwide, which resulted in a huge financial burden [5]. Unfortunately, we still do not understand the natural course of LBP, and there is no effective therapeutic approach to modify this multicause induced disease.

To search the main cause of LBP, many research groups have been concentrating on the aneural [6, 7] intervertebral disc (IVD). Since there is only sporadic nerve ending existing in the outmost layer of the annulus, IVD as the main source for LBP remains debatable [8]. However, the endplate, which is rich in nerve endings in its ossified structure [7, 9], has been overlooked. In patients with LBP, researchers have detected signal changes in the degenerative endplates by magnetic resonance imaging (MRI) [10, 11]. Moreover, the close relationship between Modic changes or endplate defects and LBP has also been verified in some previous studies [12, 13].

Endplates undergo ossification and become highly porous during IVD degeneration [14–16], and more nerve innervation occurs in degenerative endplates than in healthy endplates [17]. It has been reported that osteoclasts generated porous endplates with calcitonin gene-related peptide- (CGRP-) positive nerve ending innervation in the mice with lumbar spine instability (LSI) surgery [18]. As pain is generated by nociceptors, porous endplates with sensory nerve innervation should be the precondition for spinal pain in LSI mice.

Prostaglandin E2 (PGE2) is a lipid factor generated at the damaged region in diverse tissues, which could lead to inflammatory or neuropathic pain [19]. In the peripheral nerve system, PGE2 evokes primary sensory neurons, dorsal root ganglion (DRG), through its E-prostanoid (EP) receptors. There are 4 types of G protein-coupled EP receptors (including EP1, EP2, EP3, and EP4) mediating PGE2's function. In the previous studies, the EP4 receptor has been shown to participate in PGE2-induced inflammatory pain and sensory neuron excitability [20, 21]. In addition, selective EP4 receptor antagonists could relieve PGE2-induced inflammatory pain. For instance, it has been reported that some kinds of EP4 receptor antagonists could suppress inflammatory pain caused by carrageenan or by complete Freund's adjuvant [22–24].

The PGE2/EP4 pathway could activate a series of pain-related ion channels, such as transient receptor potential vanilloid 1 (TRPV1) [25]. TRPV1 is made up of four subunits. It is a nonselective, outwardly rectifying cation channel [26], which is distributed not only in the DRG neurons but also in the peripheral terminals [27]. Various factors could activate the TRPV1 channel, such as ligand binding [28], voltage [29], or temperature [30]. The TRPV1 channel is considered to be an aggregator of nocuous chemical, mechanical, or thermal stimuli and is demonstrated to be one of the most important ion channels participating in inflammatory or neuropathic pain [31, 32].

In this study, we found an elevated concentration of PGE2 in the porous endplate of LSI mice. This high-level PGE2 activated the TRPV1 channel in DRG neurons via its EP4 receptor in the CGRP^+^ sensory nerve, which causes spinal hypersensitivity. In particular, L161982, a selective EP4 receptor antagonist, turned down the TRPV1 current and decreased the neuronal excitability of DRG neurons to reduce spinal pain.

## 2. Materials and Methods

### 2.1. Mice and In Vivo Treatment

All animal experiments in this study were approved by the Local Committee of Animal Use and Protection of the Third Hospital of Hebei Medical University (Hebei, China). The C57BL/6J male mice were obtained from Shanghai SLAC Laboratory Animal Co. Ltd. (Shanghai, China). We anesthetized the 2-month-old mice with ketamine (at a dosage of 100 mg/kg) and xylazine (at a dosage of 10 mg/kg). For the spinous processes, supraspinous and interspinous ligaments of L3-L5 vertebrae were resected to create the LSI model that led to vertebral endplate degeneration. Correspondingly, the posterior paravertebral muscles of L3-L5 vertebrae were detached in the sham group. At 8 weeks after operation, LSI mice received vehicle or L161982 (5 mg/kg/d) (Tocris, U.S.) by intraperitoneal injection for 2 weeks. To overactivate the TRPV1 channel, LSI mice received capsaicin injection at caudal endplates of L4–L5. Specifically, 2 *μ*L capsaicin (2 mg/mL) was injected into the left part of caudal endplates of L4–L5 using borosilicate glass capillaries after drilling a hole at the left part of the endplate. The drilling holes were sealed with bone wax immediately after injection to prevent tracer leakage. After capsaicin injection, the wound was sutured, and a heating pad was used to protect mice during recovery from anesthesia. Using an overdose of isoflurane, we euthanized the animals at 4 or 8 weeks after sham or LSI operation or at 2 weeks after L161982 or vehicle treatment.

### 2.2. *μ*CT

Mice were euthanized by isoflurane and perfused by 10% buffered formalin. The L3-L5 lumbar spine was collected and examined by *μ*CT (voltage, 55 kVp; current, 181 *μ*A; 9.0 *μ*m per pixel) (Skyscan, 1176). Images were reconstructed by using NRecon v1.6 software (Skyscan). Quantitative analysis of the *μ*CT results was performed by using CTAn v1.9 software (Skyscan). Six consecutive images of the L4-L5 caudal endplates and L5 vertebrae (coronal view) were selected to show the 3-dimensional reconstruction results by using CTVol v2.0 software (Skyscan).

### 2.3. Histomorphometry and Immunofluorescence

The lumbar spine or DRG samples were dissected from mice and then were fixed in 10% buffered formalin (4°C, 24 h). The samples of the lumber spine were decalcified by 0.5 M ethylenediamine tetraacetic acid at 4°C for 3 weeks, and the L2 DRGs were dehydrated by 30% sucrose at 4°C for 48 h. The spine samples were embedded in optimal cutting temperature compound (OCT) or paraffin. The DRG samples were embedded in OCT. We used the 4 *μ*m thick sections (lumber spine) for safranin O and fast green staining. 40 *μ*m thick sections of the spine samples were used for nerve fiber-related immunostaining. 10 *μ*m thick sections of the spine or DRG sample were used for other immunostaining. For immunofluorescent staining, we incubated the sections (lumber spine or DRG) with primary antibodies to CGRP (1 : 100, Abcam, U.S.), COX2 (1 : 100, Abcam, U.S.), EP4 (1 : 100, Abcam, U.S.), and TRPV1 (1 : 200, Abcam, U.S.) (4°C, overnight). Then, we incubated the sections (lumber spine or DRG) with secondary antibodies (room temperature, 1 h, avoiding light). The fluorescence or confocal microscopes were used to capture the images of spine or DRG samples. ImageJ software (National Institutes of Health, U.S.) was used for the quantitative analysis.

### 2.4. Behavioral Testing

Pressure tolerance was measured by the vocalization thresholds (as a nociceptive threshold) using a force gauge (Bioseb). Animals were gently restrained and received the pressure force by a sensor on their skin over the L4-L5 spine. A gradual increase in pressure force (50 g/s) was performed on the mice until the animals made an audible vocalization. To prevent tissue injury, the maximum force was limited to 500 g.

Spontaneous activity was measured by several indicators (including distance traveled, active time, and maximum speed) using the activity wheels (Bioseb). Animals were kept in the cages which are similar to their home cages, and the wheels of the device could be rotated by animals in both directions. The software of this device could record the real-time data of the animals' spontaneous activity.

The pain hypersensitivity in response to mechanical stimulation was measured by hind paw withdrawal frequency (PWF) using the von Frey test with 0.07 or 0.4 g filament (Stoelting). Animals were restrained in a transparent plastic cage, which was put on a metal mesh grid. The midplantar position of the animal's hind paw was stimulated by 0.07 or 0.4 g filament through the mesh grid. The filaments should be buckled by enough pressure, and the frequency of mechanical stimulus was 10 times at a 1 s interval. When the hind paw was withdrawn after the stimulation by von Frey filaments, it was recorded.

### 2.5. Quantitative Real-Time Polymerase Chain Reaction (qRT-PCR)

The total RNA of the L4-L5 caudal endplate was extracted by using the TRIzol reagent (Tiangen, Beijing, China). We measured RNA purity by the absorbance of 260/280 nm. With the RevertAid™ First Strand cDNA Synthesis Kit (Thermo Fisher, U.S.), we reverse transcribed 1 *μ*g RNA into cDNA. Then, we performed qRT-PCR by using the SuperReal PreMix Plus (Tiangen, Beijing, China). Relative expression of target genes was analyzed by the 2^−ΔΔCT^ method. The primers used in our study are listed in Table 1.

### 2.6. Enzyme-Linked Immunosorbent Assay (ELISA)

The PGE2 Parameter Assay Kit purchased from R&D Systems (U.S.) was used to measure PGE2 concentrations in the L4-L5 endplates.

### 2.7. Western Blotting Analysis

We extracted the total protein of the L4-L5 caudal endplate by using RIPA lysis buffer (Beyotime, Shanghai, China). With 12% SDS-polyacrylamide gel electrophoresis, 20 *μ*g protein was resolved and then was transferred to polyvinylidene fluoride membranes (Millipore, U.S.). We blocked the membranes with 5% milk and incubated them with primary antibodies to EP4 (1 : 1000, Thermo Fisher, U.S.), TRPV1 (1 : 1000, Thermo Fisher, U.S.), and GAPDH (1 : 5000, Abcam, U.S.) (4°C, overnight). Then, we incubated the membranes with secondary antibodies (1 : 20,000, Rockland, U.S.) (37°C, 1 hour). Finally, with the Odyssey infrared imaging system and ImageJ software (National Institutes of Health, U.S.), the integrated intensity of the protein band was detected and analyzed, respectively.

### 2.8. Electrophysiology

As previously described, we selected the small-diameter neurons (Cm < 42 pF) for whole-cell patch clamp recording [33].

### 2.9. Voltage Clamp Recording

Pipettes (3-4 M*Ω*) were filled with the following: KCl 140, MgCl_2_ 1, CaCl_2_ 0.5, EGTA 5, HEPES 10, and ATP 3 (in mM) (pH 7.4 with KOH). The bath solution for DRG neurons was as follows: NaCl 150, KCl 5, CaCl_2_ 2.5, MgCl_2_ 1, glucose 10, and HEPES 10 (in mM) (pH 7.4 with NaOH). TRPV1 currents were acquired via an Axopatch 200B amplifier (Molecular Devices) and low passed at 5 kHz. Cells were constantly held at -60 mV, and TRPV1 currents induced by 1 *μ*M capsaicin were recorded.

### 2.10. Current Clamp Recording

The pipette solution contained the following (in mM): KCl 140, EGTA 0.5, HEPES 5, and Mg-ATP 3 (pH 7.3 with KOH). The bath solution for DRG neurons was as follows (in mM): NaCl 140, KCl 3, MgCl_2_ 2, CaCl_2_ 2, and HEPES 10 (pH 7.3 with NaOH). Cells were examined for action potential firing with a series of 1 s current from 50 pA to 500 pA in 50 pA increments or with a liner ramp of current from 0 pA to 1000 pA (500 ms duration). -200 pA (200 ms) was injected to measure membrane input resistance (*R*_in_).

### 2.11. Statistical Analysis

We conducted data analyses by using SPSS15.0 software. Data were shown as means ± standard deviations. We used unpaired two-sample *t*-test to compare the means of two groups. We used one-way ANOVA with Bonferroni's post hoc test to compare the means of multiple groups. With the two-way ANOVA with repeated measures, we analyzed the effects of LSI surgery on animals' spinal hypersensitivity and movements at different time points. We established inclusion or exclusion criteria before each experiment and did not exclude any sample during data analysis. *p* < 0.05 was regarded as the statistical significance for all experiments.

## 3. Results

### 3.1. Sensory Innervation in the Porous Endplate in LSI Mice

**Figure 1:**
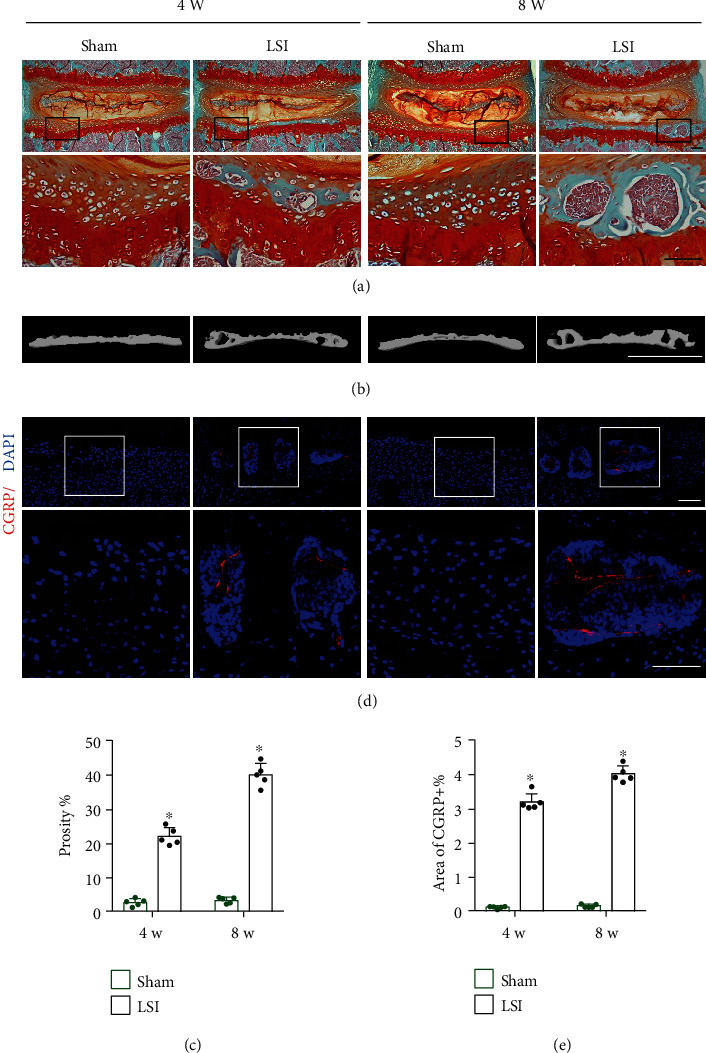
Sensory innervation in the porous endplate in LSI mice. (a) Representative images of safranin O and fast green staining of the proteoglycan (red) and bone marrow cavities (green) in the L4-L5 caudal endplates (coronal view) in the LSI or sham group. (b) Representative images of *μ*CT of the L4-L5 caudal endplates (coronal view) in the LSI or sham group. (c) Quantitative analysis of the percentage of endplate porosity examined by *μ*CT. (d) Representative images of immunostaining of CGRP (red) and DAPI (blue) in the L4-L5 caudal endplates (coronal view) in the LSI or sham group. (e) Quantitative analysis of the percentage of CGRP^+^ area in the L4-L5 caudal endplates. Scale bars, 50 *μ*m (a, d). Scale bars, 1 mm (b). ^∗^*p* < 0.05 vs. sham group at the corresponding time points. *n* = 5 per group (c, e).

To demonstrate the endplate porosity in LSI mice, we examined the L4-L5 caudal endplates after 4 and 8 weeks of surgery using histological staining and 3-dimensional *μ*CT. Safranin O and fast green staining results revealed that bone marrow cavities appeared in degenerative endplates in LSI mice, while the endplates in the sham group were cartilaginous and homogenous (Figure 1(a)). Moreover, the reconstruction of 3-dimensional *μ*CT also showed porous endplates in the LSI mice, while the microstructure of endplates was intact in the sham group (Figures 1(b) and 1(c)). However, LSI surgery did not influence the bone mass of the lumbar vertebra (Supplementary Figure 1A-E).

Immunofluorescent staining showed the innervation of CGRP^+^ nerve fibers in the porous endplate at 4 and 8 weeks after LSI surgery, but the CGRP^+^ nerve endings did not exist in homogenous endplates of sham surgery mice (Figures 1(d) and 1(e)).

### 3.2. Spinal Hypersensitivity Increased in LSI Mice

**Figure 2:**
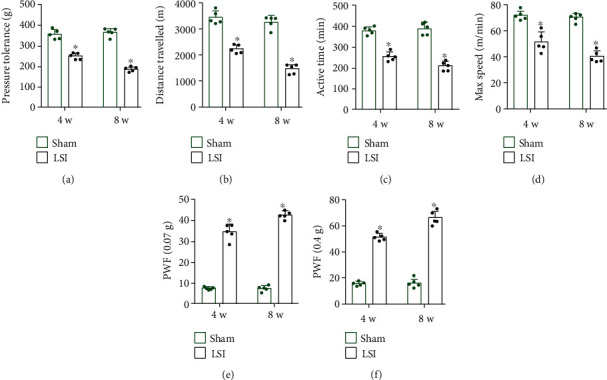
Spinal hypersensitivity increased in LSI mice. (a) Pressure tolerance was determined by a vocalization threshold in the LSI or sham group. (b–d) Voluntary and spontaneous activity was evaluated by three indicators including (b) distance traveled, (c) active time per 24 h, and (d) maximum speed of movement. (e, f) The PWF in response to the von Frey test in the LSI or sham group. ^∗^*p* < 0.05 vs. sham group at the corresponding time points. *n* = 5 per group.

In the behavior test experiments, the vocalization threshold was recorded as an indicator of pressure tolerance. We found that LSI surgery significantly decreased the pressure tolerance at 4 and 8 weeks, as compared with the sham surgery mice (Figure 2(a)).

We further examined LSI surgery effects on animals' voluntary and spontaneous activity, including distance traveled, active time per 24 h, and maximum speed of movement. All three indicators decreased significantly in LSI mice rather than in the sham group at 4 and 8 weeks (Figures 2(b)–2(d)).

Finally, we performed the von Frey test to evaluate the mechanical hypersensitivity of the hind paw, which could indirectly reflect the severity of LBP. The PWF was increased significantly by LSI surgery at 4 and 8 weeks (Figures 2(e) and 2(f)).

### 3.3. PGE2 Concentration and EP4 Expression Increased in the Porous Endplate of LSI Mice

**Figure 3:**
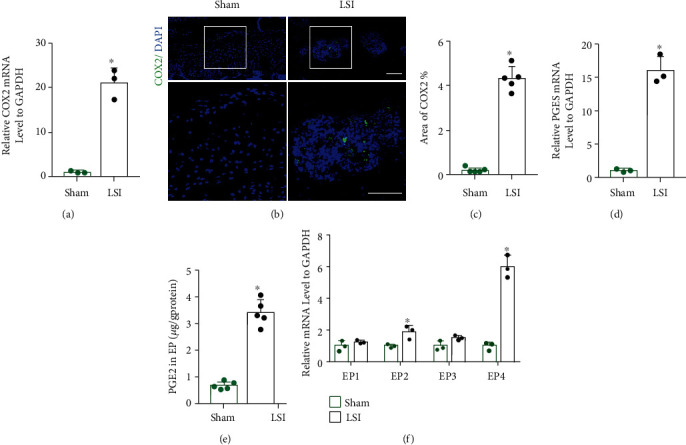
PGE2 concentration and EP4 expression increased in the porous endplate of LSI mice. (a) qRT-PCR analysis of COX-2 expression in L4-L5 caudal endplates in the LSI or sham group at 8 weeks. (b) Representative images of immunostaining of COX-2 (green) and DAPI (blue) in the L4-L5 caudal endplates in the LSI or sham group at 8 weeks. (c) Quantitative analysis of the percentage of COX-2^+^ area in endplates. (d) PGES expression by qRT-PCR in L4-L5 caudal endplates in the LSI or sham group at 8 weeks after surgery. (e) PGE2 concentration determined by ELISA analysis in L4-L5 caudal endplates in the LSI or sham group. (f) qRT-PCR analysis of EP1, EP2, EP3, and EP4 expression in L4-L5 caudal endplates in the LSI or sham group at 8 weeks after surgery. Scale bars, 50 *μ*m (b). ^∗^*p* < 0.05 vs. sham group. *n* = 3 per group (a, d, f); *n* = 5 per group (c, e).

Since PGE2 is the cyclooxygenase 2 (COX2) product in the inflammatory environment, we examined COX2 expression, prostaglandin E synthase (PGES) expression, and PGE2 concentration in L4-L5 endplates at 8 weeks in the two groups. qRT-PCR and immunostaining showed an increase in COX-2 expression at 8 weeks in the LSI group relative to the sham group (Figures 3(a)–3(c)). Similarly, PGES mRNA and PGE2 concentration was significantly increased after 8 weeks of LSI surgery in qRT-PCR and ELISA, respectively, relative to the sham group (Figures 3(d) and 3(e)).

Since there were four types of EP receptors (EP1-EP4) mediating PGE2's functions, we used qRT-PCR to evaluate the change of the mRNA levels of these four types of EP receptors after LSI surgery. Interestingly, we found a 6-fold increase in EP4 expression and a 2-fold increase in EP2 expression in the LSI group relative to the sham group by qRT-PCR. But there was no significant difference in EP1 and EP3 expression between the LSI and sham groups (Figure 3(f)).

### 3.4. EP4/TRPV1 Expressed in CGRP^+^ Nerves in the Porous Endplate and in the CGRP^+^ Neuron of L2 DRG in LSI Mice, Respectively

**Figure 4:**
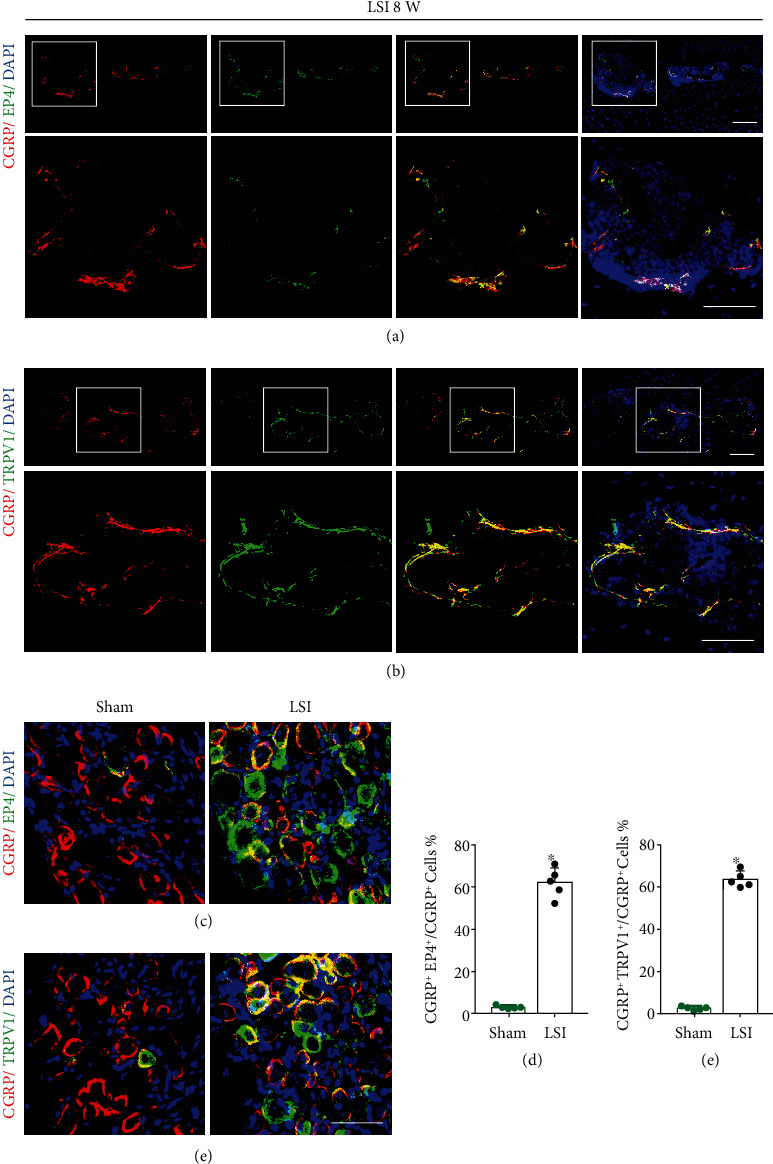
EP4 and TRPV1 expressed in CGRP^+^ nerves and in CGRP^+^ DRG neurons of LSI mice, respectively. (a) Representative images of the coimmunostaining of CGRP and EP4 in L4-L5 caudal endplates in the LSI or sham group at 8 weeks. (b) Representative images of the coimmunostaining of CGRP and TRPV1 in L4-L5 caudal endplates in the LSI or sham group at 8 weeks. (c) Representative images of the coimmunostaining of CGRP and EP4 in L2 DRGs in the LSI or sham group at 8 weeks. (d) The percentage of the EP4^+^CGRP^+^ area relative to the CGRP^+^ area in the LSI or sham group. (e) Representative images of the coimmunostaining of CGRP and TRPV1 in L2 DRGs in the LSI or sham group at 8 weeks. (f) The percentage of the TRPV1^+^CGRP^+^ area relative to the CGRP^+^ area in the LSI or sham group. Scale bars, 50 *μ*m (a, b, c, e). ^∗^*p* < 0.05 vs. sham group. *n* = 5 per group (d, f).

Immunofluorescent staining showed that EP4 expression existed in CGRP^+^ nerve fibers in degenerative endplates (Figure 4(a)). Moreover, there was also colocalization of TRPV1 and CGRP in the degenerative endplates, as examined by immunofluorescent staining (Figure 4(b)).

In a previous study, a retrograde tracing experiment was conducted in LSI mice. They found that Dil was significantly retrograded to L1-L2 DRG, especially to L2 DRG [18]. Therefore, we performed the costaining of EP4 and CGRP in L2 DRG. We found that the percentage of EP4^+^CGRP^+^/CGRP^+^ neurons was increased in the LSI group than in the sham group (Figures 4(c) and 4(d)). Meanwhile, we conducted the costaining of TRPV1 and CGRP in L2 DRG. The percentage of TRPV1^+^CGRP^+^/CGRP^+^ neurons was also increased in the LSI group (Figures 4(e) and 4(f)).

### 3.5. LSI Surgery Increased TRPV1 Channel Current Density in L2 DRG Neurons

**Figure 5:**
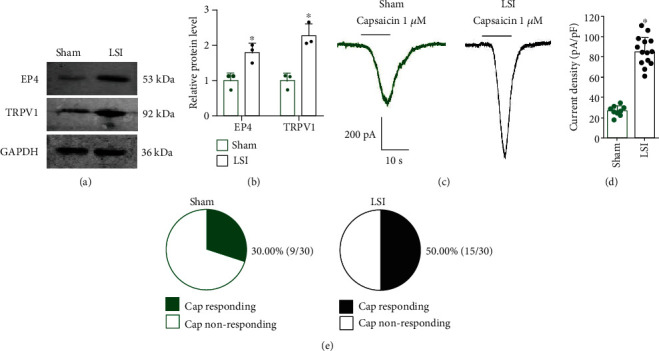
LSI surgery increased TRPV1 expression and TRPV1 channel current density in L2 DRG neurons. (a) Representative images of western blotting of EP4 and TRPV1 expression in L2 DRGs in the LSI or sham group at 8 weeks after surgery. (b) Quantitative analysis of EP4 and TRPV1 expression in L2 DRGs in the LSI or sham group at 8 weeks after surgery (*n* = 3 per group). (c) Representative traces of TRPV1 current induced by 1 *μ*M capsaicin. (d) The TRPV1 current amplitude induced by 1 *μ*M capsaicin increased significantly in LSI mice (*n* = 9-15 cells per group). (e) The number of 1 *μ*M capsaicin-responding neurons was increased after LSI treatment (*n* = 30 cells per group). ^∗^*p* < 0.05 vs. sham group.

Western blotting analysis showed that EP4 and TRPV1 expression increased in L2 DRG in LSI mice compared with the sham group (Figures 5(a) and 5(b)).

L2 DRG neurons were isolated from the mice at 8 weeks and then were cultured overnight. With the whole-cell patch clamp, we did the electrophysiological experiments in small-size neurons (Cm < 42 pF) taken from L2 DRGs [33]. The TRPV1 current amplitude (1 *μ*M capsaicin) increased significantly in LSI mice (Figures 5(c) and 5(d)). Furthermore, the proportion of capsaicin-responsive neurons also increased in LSI mice relative to the sham group (Figure 5(e)).

### 3.6. L161982, a Selective EP4 Receptor Antagonist, Reduced Spinal Hypersensitivity in LSI Mice

**Figure 6:**
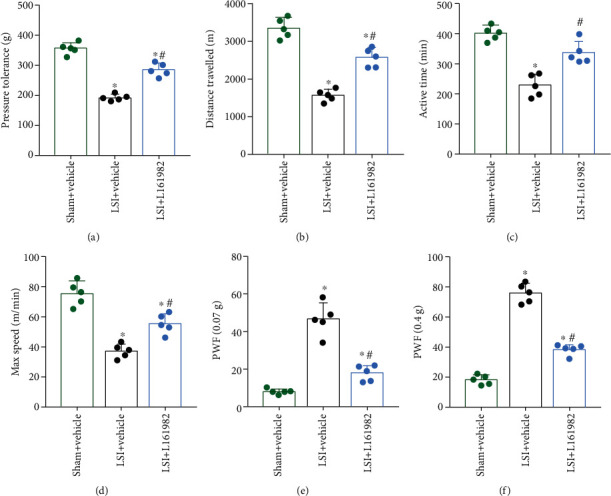
L161982 reduced spinal hypersensitivity of LSI mice. (a) Pressure tolerance was determined by a vocalization threshold at 2 weeks after L161982 or vehicle treatment. (b–d) Voluntary and spontaneous activity was evaluated by three indicators including (b) distance traveled, (c) active time per 24 h, and (d) maximum speed of movement. (e, f) The PWF in response to the von Frey test (0.07 g or 0.4 g) at 2 weeks after L161982 or vehicle treatment. ^∗^*p* < 0.05 vs. sham+vehicle group; ^#^*p* < 0.05 vs. LSI+vehicle group. *n* = 5 per group.

We used L161982, an EP4-receptor antagonist, to investigate the effects of blocking PGE2/EP4 signaling on spinal hypersensitivity. In pressure tolerance and spontaneous activity tests, L161982 treatment increased pressure tolerance and spontaneous activity of LSI mice compared to the vehicle group (Figures 6(a)–6(d)).

Similarly, the inhibitory effect of L161982 on hind paw mechanical hypersensitivity, as indicated by decreased PWF to 0.07 g or 0.4 g stimulation, was also demonstrated at 2 weeks after treatment (Figures 6(e) and 6(f)).

However, the EP4 receptor antagonist L161982 did not influence the endplate porosity of LSI mice (Supplementary Figure 1F, G).

Moreover, we injected capsaicin at the caudal endplate of L4-L5 of LSI mice to overactivate the TRPV1 channel. We found that TRPV1 overactivation increased spinal hypersensitivity based on the behavior test results. And the spinal hypersensitivity was obviously increased in the LSI+capsaicin+L161982 group, compared with the LSI+L161982 group (Supplementary Figure 2A-F).

### 3.7. L161982 Reduced TRPV1 Channel Current Density in L2 DRG Neurons

**Figure 7:**
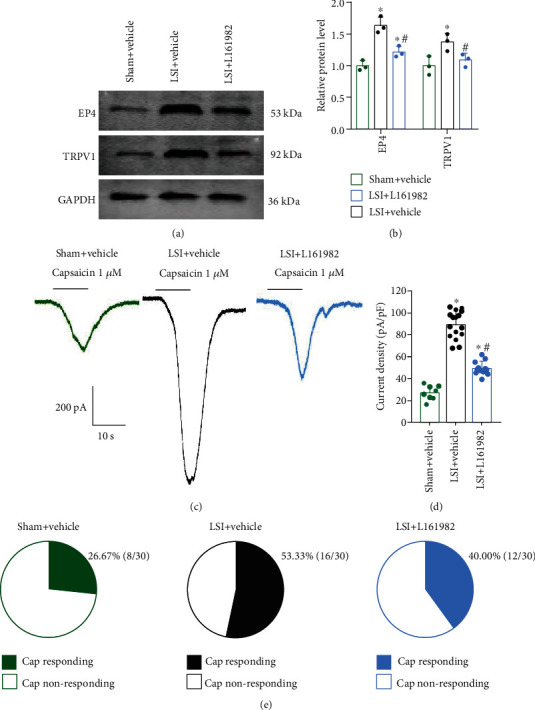
L161982 reduced TRPV1 expression and TRPV1 channel current density in L2 DRG neurons of LSI mice. (a) Representative images of western blotting of EP4 and TRPV1 expression in L2 DRGs at 2 weeks in the sham+vehicle, LSI+vehicle, and LSI+L161982 group. (b) Quantitative analysis of EP4 and TRPV1 expression in L2 DRGs at 2 weeks in the sham+vehicle, LSI+vehicle, and LSI+L161982 group (*n* = 3 per group). (c) Representative traces of TRPV1 current induced by 1 *μ*M capsaicin. (d) Quantitative analysis of 1 *μ*M capsaicin-induced current densities (*n* = 8-16 cells per group). (e) The percentage of the neurons in response to 1 *μ*M capsaicin (*n* = 30 cells per group). ^∗^*p* < 0.05 vs. sham+vehicle group, ^#^*p* < 0.05 vs. LSI+vehicle group.

Western blotting analysis showed that EP4 and TRPV1 expression decreased in L2 DRG of mice with L161982 treatment relative to vehicle treatment (Figures 7(a) and 7(b)).

The TRPV1 current amplitude (1 *μ*M capsaicin) decreased significantly in LSI mice with L161982 treatment relative to vehicle treatment (Figures 7(c) and 7(d)).

In addition, the capsaicin-responsive neuron percentage decreased in the L161982 group compared to the vehicle group (Figure 7(e)).

We found that TRPV1 overactivation by capsaicin injection increased TRPV1 current measured with a patch clamp. And the TRPV1 current was obviously increased in the LSI+capsaicin+L161982 group, compared with the LSI+L161982 group (Supplementary Figure 3A, B).

### 3.8. L161982 Reduces the Excessive Neuronal Excitability of DRG Neurons Induced by LSI

To determine whether LSI surgery increases DRG neuronal excitability and whether PGE2/EP4/TRPV1 pathway activation is responsible for DRG neuron hyperexcitability of LSI mice, evoked action potentials (APs) were studied by current clamp recording.

**Figure 8:**
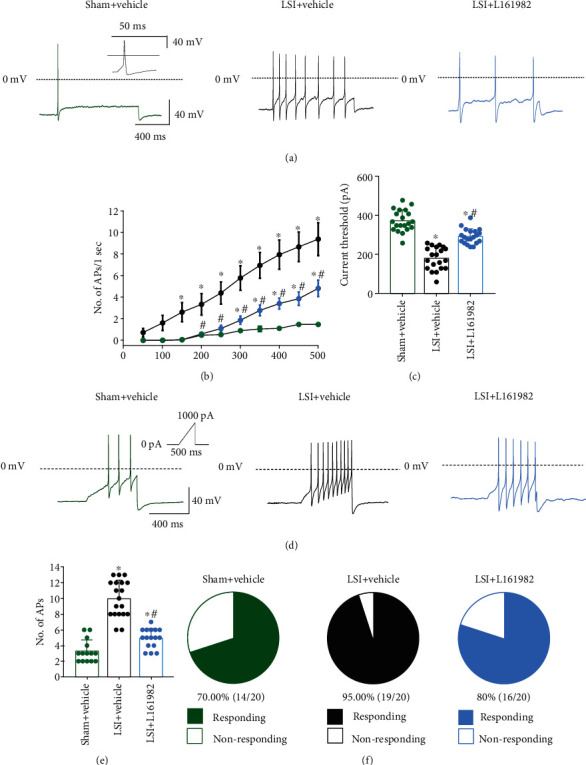
L161982 treatment attenuated LSI-induced increase in neuronal excitability. (a) AP firing traces in L2 DRG neurons to 1 s, 300 pA depolarizing current injection. (b) Quantitative analysis of APs induced by step current injection in the sham+vehicle, LSI+vehicle, and LSI+L161982 groups. (c) Current threshold for APs in the sham+vehicle, LSI+vehicle, and LSI+L161982 groups (*n* = 20 cells per group). (d) Current clamp recordings with ramp current stimulation starting from 0 pA to 1000 pA of 500 ms duration. (e) Quantitative analysis of APs induced by ramp current stimulation (*n* = 14-19 cells per group). (f) The percentage of the neurons in response to ramp current stimulation (*n* = 20 cells per group). ^∗^*p* < 0.05 vs. sham+vehicle group, ^#^*p* < 0.05 vs. LSI+vehicle group.

With step current injection, LSI surgery increased AP firing frequency compared to the sham group, and the AP firing frequency could be reduced by L161982 treatment (Figures 8(a) and 8(b) and Table 2). The minimal depolarizing current that could evoke APs was significantly decreased after LSI operation, which could also be reversed by L161982 (Figure 8(c) and Table 2).

In addition, we evaluated the neuronal hyperexcitability by ramp current stimulation. LSI surgery significantly increased the firing of APs relative to the sham group, and the firing of APs was lowered by L161982 treatment (Figures 8(d) and 8(e) and Table 2). The percentage of neurons which fired APs was also calculated under the simulation of ramp current injection. We found a higher responding rate in LSI mice compared with the sham group, and the responding rate was significantly lowered by L161982 treatment (Figure 8(f)).

## 4. Discussion

The IVD degeneration is regarded as one of the most common diseases causing LBP [34]. In recent decades, Modic changes, manifested as signal changes in endplates by MRI, have been demonstrated to be a specific cause of LBP [35]. Endplates undergo ossification and become porous during IVD degeneration, which leads to LBP [36, 37]. It has been reported that more nerve innervation occurs in degenerative endplates than in healthy endplates [17]. In our study, we used a mouse model of vertebral endplate degeneration by LSI surgery [14]. According to behavior test experiments, the pressure tolerance and spontaneous activity significantly decreased in LSI mice, whereas the hind paw mechanical hypersensitivity significantly increased in this model.

Consistent with the previous study [18], we demonstrated that CGRP^+^ nerves innervated in the porous endplate of LSI mice. It has been reported that CGRP could be generated from peripheral or central nerve fibers as the mechanical stimuli on skin [38]. CGRP receptors are demonstrated to be widely distributed in the pain-related pathway [39]. Acute or chronic nociception could promote sensory nerves or central terminals to generate more CGRP into the dorsal horn [40, 41]. Thus, the CGRP^+^ nerve innervated in the porous endplate, which was the precondition for spinal hypersensitivity in LSI mice.

In our study, we found that COX2 expression and PGE2 concentration were significantly increased in the porous endplate in LSI mice. Moreover, there was a 6-fold increase in EP4 expression and a 2-fold increase in EP2 expression in the endplate of LSI mice relative to sham mice, but there was no significant difference in EP1 and EP3 expression between the two groups. Thus, the PGE2/EP4 pathway might play a crucial role in spinal hypersensitivity of this animal model. When tissue was damaged, the inflammatory mediators, such as PGE2, were released at the local region or in the spinal cord [42]. PGE2 induces pain sensitization and leads to CGRP release in sensory nerves in vivo [43], as well as in cultured DRG neurons in vitro [44]. PGE2 displays functions via its G protein-coupled receptors (EP1–EP4) [45]. The EP4 receptor is coupled with G protein and activates adenylate cyclase, which enhances the intracellular activation of cAMP-dependent protein kinases (e.g., PKA) [46]. PGE2 has been reported to promote the capsaicin-evoked CGRP generation by DRG neurons via its G protein-coupled EP receptor, EP4 receptor [21]. In our study, we demonstrated the colocalization of EP4 and CGRP in the nerve endings both in porous endplates and in the DRG neurons. Besides, we also found the colocalization of TRPV1 and CGRP in the nerve endings both in porous endplates and in the DRG neurons by immunofluorescent staining.

The crucial role of TRPV1 activation in spinal pain of LSI mice was also demonstrated in our present study. We found a higher expression of TRPV1 in L2 DRG which innervated in L4-L5 endplates of LSI mice. The upregulated expression of TRPV1 in L2 DRG correlated well with the increase in spinal hypersensitivity. Furthermore, the patch clamp results showed that LSI operation increased TRPV1 current density, suggesting that the functional TRPV1 expression was increased by LSI surgery. Thus, the increased current density of the TRPV1 channel might participate in LSI-induced spinal hypersensitivity.

TRPV1, a member of TRP ion channels, has been recognized as “a molecular gateway” to nociceptive sensation. TRPV1 was mainly distributed in the dorsal root ganglion, trigeminal ganglion, spinal cord, and peripheral nerve endings. In addition, TRPV1 was also found in some nonneural tissues such as the lung, gastrointestinal tract, and respiratory tract. In recent years, it has been found that TRPV1 is important in mediating hypersensitivity mediated by inflammation nocuous chemical, mechanical, or thermal stimuli in the airway, skin, gastrointestinal tract, and other organs [47–51]. There is less evidence about TRPV1-mediating hypersensitivity in a vertebral endplate degeneration model. However, in the arthritis model, whose pathogenesis is similar to the vertebral endplate degeneration model, the fact that TRPV1 is important in mediating hypersensitivity has been proven. Thermal hyperalgesia and osteoarthritic pain are associated with the activation of the TRPV1 channel [52]. TRPV1 may contribute to the pain hypersensitivity and inflammation of arthritis via an ERK-mediated pathway [53]. Polypeptide APHC3, a mode-selective TRPV1 antagonist, can significantly reverse mechanical hypersensitivity in the arthritis model [54]. The above evidence shows that TRPV1 is important in mediating hypersensitivity in degenerative osteoarthritis.

TRPV1 contributes to spinal hypersensitivity. Evidence proved that hypersensitivity induced by activation of spinal cord PAR2 receptors is mediated by TRPV1 receptors [55]. TRPV1 was functionally expressed in GABAergic spinal interneurons, and activation of spinal TRPV1 resulted in long-term depression of excitatory inputs and a reduction of inhibitory signaling to spinothalamic tract projection neurons and eventually leads to central sensitization [56]. Evidence has demonstrated that blocking TRPV1 could relieve spinal hypersensitivity. The thermal and mechanical hypersensitivity in the spine can be relieved by the TRPV1 selective antagonist [57]. Intrathecal administration of the antisense oligonucleotide against TRPV1 reduced mechanical hypersensitivity in rats with spinal nerve ligation [58]. The hypersensitivity induced by lumbar 4 spinal nerve ligation in mice was completely reversed by the TRPV1 antagonist A-425619 [59]. The threshold against heat sensitivity in the L5 ipsilateral dorsal horn of the spinal cord was markedly prolonged in Trpv1-/- mice than in WT mice [60]. Capsazepine, a TRPV1 blocker, could greatly inhibit thermal hypersensitivity in a spinally sensitized state [61]. AMG9810, the specific antagonist of TRPV1, could significantly attenuate the activation of bilateral spinal astrocytes and microglia [33]. The above evidence indicates that blocking TRPV1 could relieve spinal hypersensitivity.

Actually, there is a close relationship between the PGE2/EP4 pathway and TRPV1 channel. PGE2 has been shown to increase surface trafficking of EP4 and TRPV1 in vitro [62]. In a restraint stress rat model, overproduced PGE2 in injured nerves chronically increased EP4 and TRPV1 expression in primary sensory neurons, and EP4 antagonists relieved both inflammatory and neuropathic pain [25]. In our study, using behavior test experiments, we found that L161982, an EP4 receptor antagonist, relieved spinal hypersensitivity by blocking the PGE2/EP4 pathway in LSI mice. Furthermore, L161982 decreased the TRPV1 current density and the proportion of capsaicin-responsive neurons relative to L2 DRG neurons in LSI mice.

PGE2 acts on target cells through its receptors EP1, EP2, EP3, and EP4. Interactions of PGE2/EP4 and TRPV1 in pain hypersensitivity have been proven. PGE2 enhanced capsaicin-induced currents in DRG neurons through EP4 [20] and EP4-PKA signaling cascades [63]. PGE2 potentiated pain evoked by the TRPV1 agonist [64]. The upregulation of TRPV1 in DRG neurons was suppressed by a selective COX2 inhibitor, suggesting that PGE2 stimulates TRPV1 synthesis in DRG neurons [65]. Furthermore, PGE2-induced thermal hyperalgesia was abolished in TRPV1-knockout mice [63]. The above evidences suggest that functional interactions between PGE2/EP4 and TRPV1 are crucial to PGE2-induced nociceptor sensitization. A recent study has proven that PGE2/EP4 increased TRPV1 cell surface trafficking in DRG neurons via cAMP/PKA/ERK/MAPK signaling pathways. Moreover, PGE2 induced TRPV1 externalization and enhances TRPV1 activity [62].

In our study, we showed that L2 DRG neurons exhibited an increased excitability in the LSI model. The hyperexcitability of DRG neurons was decreased by the inhibition of the PEG2/EP4 pathway with L161982. These results showed that TRPV1 channel activated by the PEG2/EP4 pathway participated in the enhancement of the excitability of DRG neurons in LSI mice. It has been reported that the hyperexcitability of DRG neurons leads to central sensitization and chronic pain [66]. Therefore, the TRPV1 channel activated by the PEG2/EP4 pathway caused the hyperexcitability of DRG neurons, which could drive spinal pain.

In conclusion, the PGE2/EP4 pathway in the porous endplate could activate the TRPV1 channel in DRG neurons to cause spinal hypersensitivity in LSI mice. L161982, a selective EP4 receptor antagonist, could turn down the TRPV1 current and decrease the neuronal excitability in DRG neurons to reduce spinal pain.

## Figures and Tables

**Table 1:** The primer sequence for qRT-PCR.

Target gene	Forward primer	Reverse primer
COX2	CAGACAACATAAACTGCGCCTT	GATACACCTCTCCACCAATGACC
PGES	TTTCTGCTCTGCAGCACACT	GATTGTCTCCATGTCGTTGC
EP1	GACGATTCCGAAAGACCGCAG	CAACACCACCAACACCAGCAG
EP2	GATGGCAGAGGAGACGGAC	ACTGGCACTGGACTGGGTAGA
EP3	TGCTGGCTCTGGTGGTGAC	ACTCCTTCTCCTTTCCCATCTGTG
EP4	CTGGTGGTGCTCATCTGCTC	AGGTGGTGTCTGCTTGGGTC
GAPDH	AATGTGTCCGTCGTGGATCTGA	AGTGTAGCCCAAGATGCCCTTC

**Table 2:** Summary of current clamp properties of DRG neurons.

Current clamp properties	Sham+vehicle			LSI+vehicle			LSI+L161982		
	Mean	SD	*n*	Mean	SD	*n*	Mean	SD	*n*
Input resistance (M*Ω*)	520.91	154.55	20	521.60	153.04	20	497.21	172.56	20
Capacitance (pF)	22.32	4.88	20	22.46	4.92	20	23.50	3.96	20
RMP (mV)	-59.00	7.57	20	-58.50	6.64	20	-58.65	7.39	20
AP amplitude (mV)	112.90	9.94	20	114.43	10.03	20	113.20	6.60	20
Threshold (pA, ramp protocol)	470.00	134.54	14	204.26^∗^	81.77	19	351.38^∗^^#^	71.45	16

^∗^*p* < 0.05 vs. sham+vehicle group, ^#^*p* < 0.05 vs. LSI+vehicle group.

## Data Availability

The data used to support the findings of this study are available from the corresponding author upon request.

## Supplementary Materials

Supplementary Materials Supplementary Figure 1: the effects of LSI treatment on vertebra bone mass and L161982 treatment on endplate porosity. (A) Representative three-dimensional high-resolution *μ*CT images of the trabecular bone of the L5 vertebra (coronal view) at 8 weeks after sham or LSI surgery. (B–E) Quantitative analysis of the trabecular bone volume/total volume (BV/TV, B) and trabecular bone number (Tb.N, C), trabecular bone thickness (Tb.Th, D), and trabecular bone separation distribution (Tb.Sp, E) in the L5 vertebra determined by *μ*CT. ^∗^*p* < 0.05 vs. sham group. *n* = 5 per group. (F) Representative images of *μ*CT of the L4-L5 caudal endplates (coronal view) in the sham+vehicle, LSI+vehicle, or LSI+L161982 group. (G) Quantitative analysis of the percentage of endplate porosity examined by *μ*CT. ^∗^*p* < 0.05 vs. sham+vehicle group, ^#^*p* < 0.05 vs. LSI+vehicle group. *n* = 5 per group. Supplementary Figure 2: spinal hypersensitivity increased by TRPV1 overactivation. (A) Pressure tolerance was determined by the vocalization threshold in the LSI+vehicle, LSI+L161982, LSI+capsaicin, or LSI+L161982+capsaicin group. (B–D) Voluntary and spontaneous activity was evaluated by three indicators including (B) distance traveled, (C) active time per 24 h, and (D) maximum speed of movement. (E, F) The PWF in response to the von Frey test (0.07 g or 0.4 g) in the LSI+vehicle, LSI+L161982, LSI+capsaicin, or LSI+L161982+capsaicin group. ^∗^*p* < 0.05 vs. LSI+vehicle group, ^#^*p* <0.05 vs. LSI+L161982 group, ^†^*p* < 0.05 vs. LSI+capsaicin group. *n* = 5 per group. Supplementary Figure 3: TRPV1 channel current density in L2 DRG neurons by TRPV1 overactivation. (A) Representative traces of TRPV1 current induced by 1 *μ*M capsaicin. (B) Quantitative analysis of 1 *μ*M capsaicin-induced current densities (*n* = 11-15 cells per group). ^∗^*p* < 0.05 vs. LSI+vehicle group, ^#^*p* < 0.05 vs. LSI+L161982 group, ^†^*p* < 0.05 vs. LSI+capsaicin group.
