# Supplementary figures and images for: TRPV1 Channel Activated by the PGE2/EP4 Pathway Mediates Spinal Hypersensitivity in a Mouse Model of Vertebral Endplate Degeneration
Source: Oxid Med Cell Longev. 2021 Aug 21;2021:9965737. doi: 10.1155/2021/9965737 (PMC8405310; doi:10.1155/2021/9965737)

Supplementary Figure 1

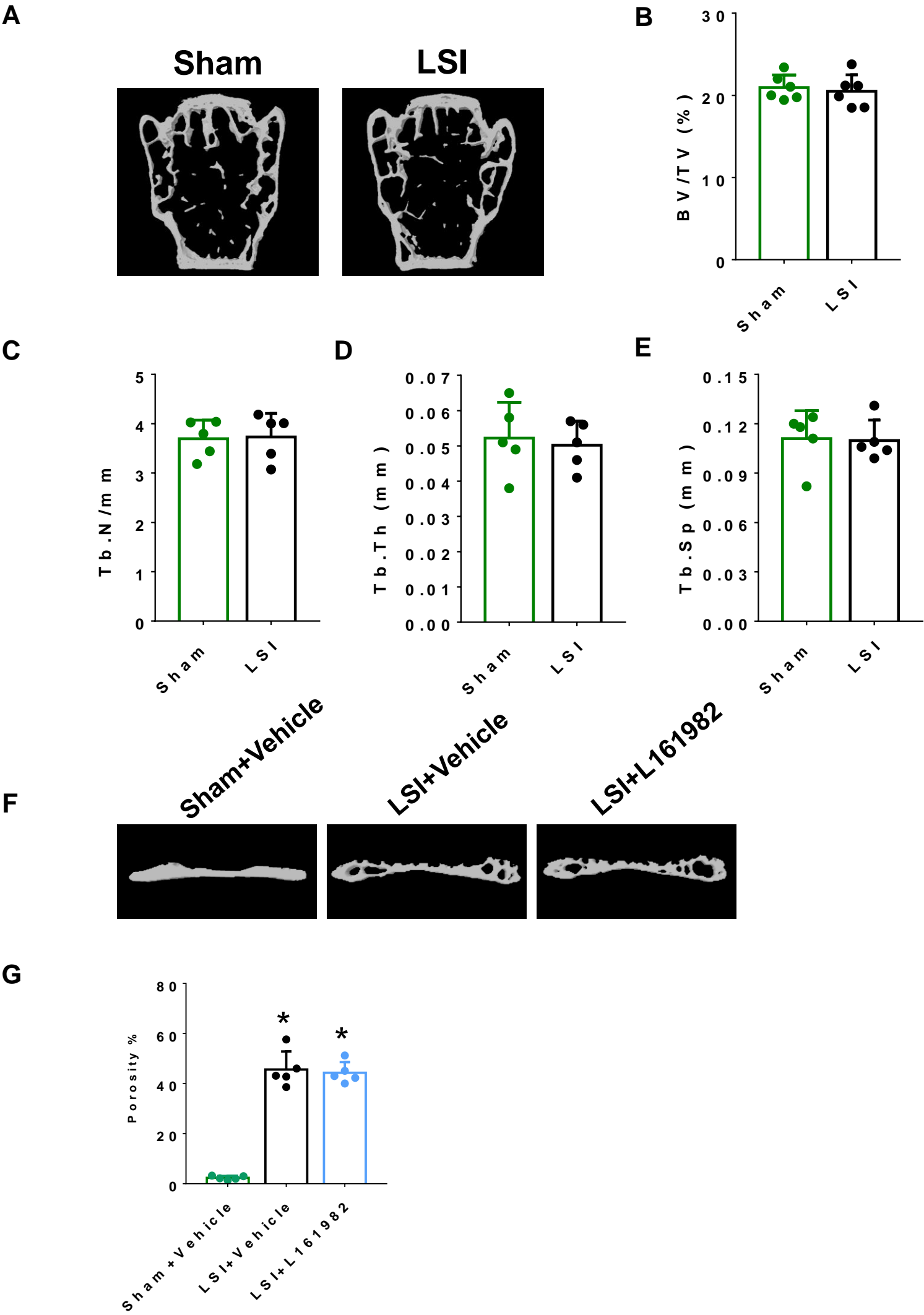

Supplement: Supplementary Materials — Supplementary Figure 1: the effects of LSI treatment on vertebra bone mass and L161982 treatment on endplate porosity. (A) Representative three-dimensional high-resolution μCT images of the trabecular bone of the L5 vertebra (coronal view) at 8 weeks after sham or LSI surgery. (B–E) Quantitative analysis of the trabecular bone volume/total volume (BV/TV, B) and trabecular bone number (Tb.N, C), trabecular bone thickness (Tb.Th, D), and trabecular bone separation distribution (Tb.Sp, E) in the L5 vertebra determined by μCT. ∗p < 0.05 vs. sham group. n = 5 per group. (F) Representative images of μCT of the L4-L5 caudal endplates (coronal view) in the sham+vehicle, LSI+vehicle, or LSI+L161982 group. (G) Quantitative analysis of the percentage of endplate porosity examined by μCT. ∗p < 0.05 vs. sham+vehicle group, #p < 0.05 vs. LSI+vehicle group. n = 5 per group. Supplementary Figure 2: spinal hypersensitivity increased by TRPV1 overactivation. (A) Pressure tolerance was determined by the vocalization threshold in the LSI+vehicle, LSI+L161982, LSI+capsaicin, or LSI+L161982+capsaicin group. (B–D) Voluntary and spontaneous activity was evaluated by three indicators including (B) distance traveled, (C) active time per 24 h, and (D) maximum speed of movement. (E, F) The PWF in response to the von Frey test (0.07 g or 0.4 g) in the LSI+vehicle, LSI+L161982, LSI+capsaicin, or LSI+L161982+capsaicin group. ∗p < 0.05 vs. LSI+vehicle group, #p <0.05 vs. LSI+L161982 group, †p < 0.05 vs. LSI+capsaicin group. n = 5 per group. Supplementary Figure 3: TRPV1 channel current density in L2 DRG neurons by TRPV1 overactivation. (A) Representative traces of TRPV1 current induced by 1 μM capsaicin. (B) Quantitative analysis of 1 μM capsaicin-induced current densities (n = 11-15 cells per group). ∗p < 0.05 vs. LSI+vehicle group, #p < 0.05 vs. LSI+L161982 group, †p < 0.05 vs. LSI+capsaicin group. [file 9965737.f1.zip › 9965737.f1.pdf]

Supplementary Figure 2

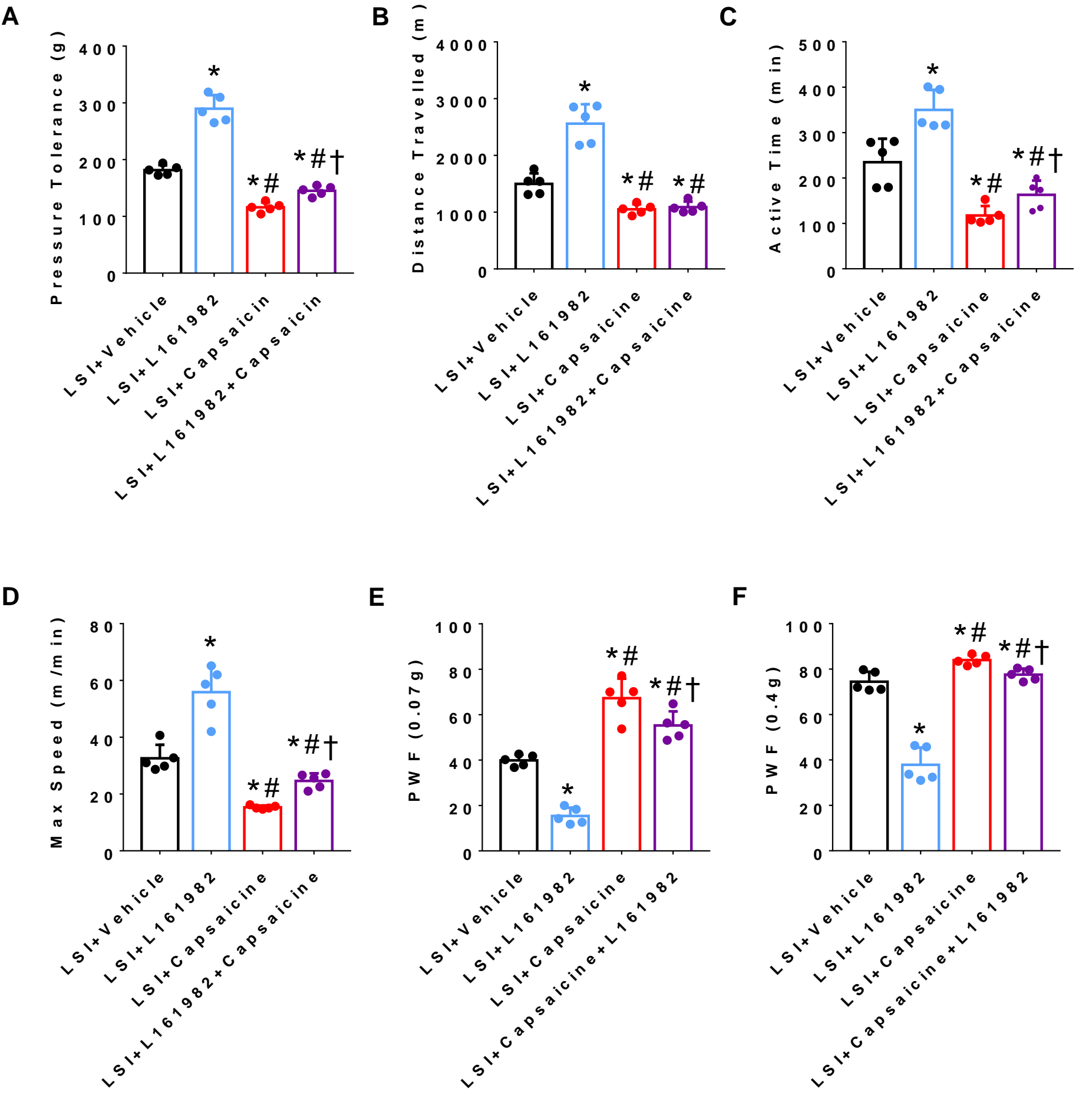

Supplement: Supplementary Materials — Supplementary Figure 1: the effects of LSI treatment on vertebra bone mass and L161982 treatment on endplate porosity. (A) Representative three-dimensional high-resolution μCT images of the trabecular bone of the L5 vertebra (coronal view) at 8 weeks after sham or LSI surgery. (B–E) Quantitative analysis of the trabecular bone volume/total volume (BV/TV, B) and trabecular bone number (Tb.N, C), trabecular bone thickness (Tb.Th, D), and trabecular bone separation distribution (Tb.Sp, E) in the L5 vertebra determined by μCT. ∗p < 0.05 vs. sham group. n = 5 per group. (F) Representative images of μCT of the L4-L5 caudal endplates (coronal view) in the sham+vehicle, LSI+vehicle, or LSI+L161982 group. (G) Quantitative analysis of the percentage of endplate porosity examined by μCT. ∗p < 0.05 vs. sham+vehicle group, #p < 0.05 vs. LSI+vehicle group. n = 5 per group. Supplementary Figure 2: spinal hypersensitivity increased by TRPV1 overactivation. (A) Pressure tolerance was determined by the vocalization threshold in the LSI+vehicle, LSI+L161982, LSI+capsaicin, or LSI+L161982+capsaicin group. (B–D) Voluntary and spontaneous activity was evaluated by three indicators including (B) distance traveled, (C) active time per 24 h, and (D) maximum speed of movement. (E, F) The PWF in response to the von Frey test (0.07 g or 0.4 g) in the LSI+vehicle, LSI+L161982, LSI+capsaicin, or LSI+L161982+capsaicin group. ∗p < 0.05 vs. LSI+vehicle group, #p <0.05 vs. LSI+L161982 group, †p < 0.05 vs. LSI+capsaicin group. n = 5 per group. Supplementary Figure 3: TRPV1 channel current density in L2 DRG neurons by TRPV1 overactivation. (A) Representative traces of TRPV1 current induced by 1 μM capsaicin. (B) Quantitative analysis of 1 μM capsaicin-induced current densities (n = 11-15 cells per group). ∗p < 0.05 vs. LSI+vehicle group, #p < 0.05 vs. LSI+L161982 group, †p < 0.05 vs. LSI+capsaicin group. [file 9965737.f1.zip › 9965737.f2.pdf]

Supplementary Figure 3

A

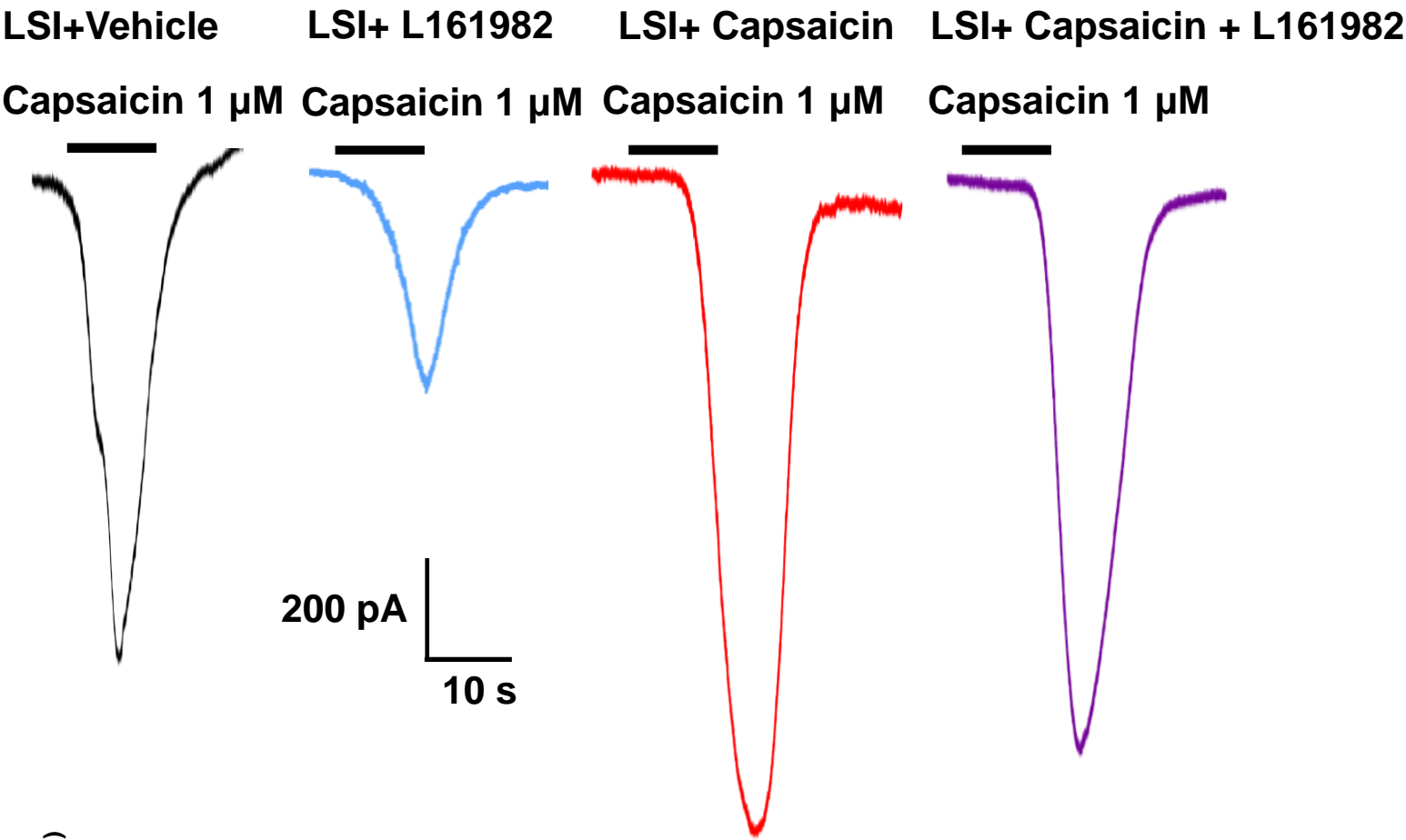

B

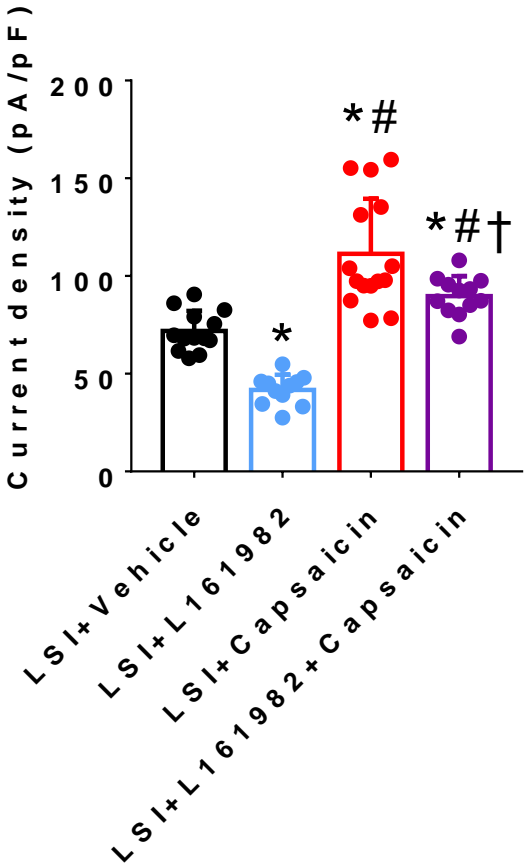

Supplement: Supplementary Materials — Supplementary Figure 1: the effects of LSI treatment on vertebra bone mass and L161982 treatment on endplate porosity. (A) Representative three-dimensional high-resolution μCT images of the trabecular bone of the L5 vertebra (coronal view) at 8 weeks after sham or LSI surgery. (B–E) Quantitative analysis of the trabecular bone volume/total volume (BV/TV, B) and trabecular bone number (Tb.N, C), trabecular bone thickness (Tb.Th, D), and trabecular bone separation distribution (Tb.Sp, E) in the L5 vertebra determined by μCT. ∗p < 0.05 vs. sham group. n = 5 per group. (F) Representative images of μCT of the L4-L5 caudal endplates (coronal view) in the sham+vehicle, LSI+vehicle, or LSI+L161982 group. (G) Quantitative analysis of the percentage of endplate porosity examined by μCT. ∗p < 0.05 vs. sham+vehicle group, #p < 0.05 vs. LSI+vehicle group. n = 5 per group. Supplementary Figure 2: spinal hypersensitivity increased by TRPV1 overactivation. (A) Pressure tolerance was determined by the vocalization threshold in the LSI+vehicle, LSI+L161982, LSI+capsaicin, or LSI+L161982+capsaicin group. (B–D) Voluntary and spontaneous activity was evaluated by three indicators including (B) distance traveled, (C) active time per 24 h, and (D) maximum speed of movement. (E, F) The PWF in response to the von Frey test (0.07 g or 0.4 g) in the LSI+vehicle, LSI+L161982, LSI+capsaicin, or LSI+L161982+capsaicin group. ∗p < 0.05 vs. LSI+vehicle group, #p <0.05 vs. LSI+L161982 group, †p < 0.05 vs. LSI+capsaicin group. n = 5 per group. Supplementary Figure 3: TRPV1 channel current density in L2 DRG neurons by TRPV1 overactivation. (A) Representative traces of TRPV1 current induced by 1 μM capsaicin. (B) Quantitative analysis of 1 μM capsaicin-induced current densities (n = 11-15 cells per group). ∗p < 0.05 vs. LSI+vehicle group, #p < 0.05 vs. LSI+L161982 group, †p < 0.05 vs. LSI+capsaicin group. [file 9965737.f1.zip › 9965737.f3.pdf]
